# Supplementary material for: Inadequate structural constraint on Fab approach rather than paratope elicitation limits HIV-1 MPER vaccine utility
Source: Nat Commun. 2023 Nov 8;14:7218. doi: 10.1038/s41467-023-42097-6 (PMC10632514; doi:10.1038/s41467-023-42097-6)
Supplement: Supplementary file 3 — Reporting Summary [file 41467_2023_42097_MOESM3_ESM.pdf]

nature portfolio

Corresponding author(s): Mikyung Kim, Ellis L. Reinherz
DAPR, NCOMMS-23-18324

Last updated by author(s): Aug 31, 2023

Reporting Summary

Nature Portfolio wishes to improve the reproducibility of the work that we publish. This form provides structure for consistency and transparency in reporting. For further information on Nature Portfolio policies, see our Editorial Policies and the Editorial Policy Checklist.

Statistics

For all statistical analyses, confirm that the following items are present in the figure legend, table legend, main text, or Methods section.

n/a	Confirmed	
[ ]	[x]	The exact sample size (*n*) for each experimental group/condition, given as a discrete number and unit of measurement
[ ]	[x]	A statement on whether measurements were taken from distinct samples or whether the same sample was measured repeatedly
[ ]	[x]	The statistical test(s) used AND whether they are one- or two-sided<br>*Only common tests should be described solely by name; describe more complex techniques in the Methods section.*
[x]	[ ]	A description of all covariates tested
[x]	[ ]	A description of any assumptions or corrections, such as tests of normality and adjustment for multiple comparisons
[ ]	[x]	A full description of the statistical parameters including central tendency (e.g. means) or other basic estimates (e.g. regression coefficient) AND variation (e.g. standard deviation) or associated estimates of uncertainty (e.g. confidence intervals)
[ ]	[x]	For null hypothesis testing, the test statistic (e.g. *F*, *t*, *r*) with confidence intervals, effect sizes, degrees of freedom and *P* value noted<br>*Give P values as exact values whenever suitable.*
[x]	[ ]	For Bayesian analysis, information on the choice of priors and Markov chain Monte Carlo settings
[x]	[ ]	For hierarchical and complex designs, identification of the appropriate level for tests and full reporting of outcomes
[x]	[ ]	Estimates of effect sizes (e.g. Cohen's *d*, Pearson's *r*), indicating how they were calculated

Our web collection on statistics for biologists contains articles on many of the points above.

Software and code

Policy information about availability of computer code

Data collection

-X-ray diffraction data were collected at 100 K from cryocooled crystals at the 19-ID beamline of the Structural Biology Center at the Advanced Photon Source at Argonne National Laboratory.

-Molecular models of IgG1 and IgG3 in Fig 6A and Supplementary Fig 15A were generated using CHARMM (https://secure-web.cisco.com/13ejXTY85kfGHjt6Kolz0e5HM4AVCKFTirX1zbnBZgBw96EuPwVCta1n9EgveF8v6QUqZR_RaIs65s8hMUI-BoaFjedHvCkZun2TpFJHOPBuI8Jl_JmvbuHzLFr7hSbN1b9e0ATsZqzUmku9SJUd9HtBJ_JSjI09BKi-KpqB_r7rrtRgK8LNbkMhLQy5I5zV44L_h_6hYyhhp4l-HHn2535dbWhZyC7VSGuhQdyD90llDd89ksAY_RhEM6o1kW7p5bON6UyDKuAEL7s780py6lA4bvHZGK3ZAFq-R_SXOw9toJO5Jmw4JBR4O5axAL0K7/https%3A%2F%2Facademiccharmm.org%2F).

-C++ codes together with instructions needed to generate the virion models in Fig 6B and Supplementary Fig 15B are in: https://secure-web.cisco.com/1JMfRTa5aTYYNEIM4OWGGbtymhtKCwjJPZhF86UjAGxE-mSxevGMdnicgyJTCZG07vO0SPEzyhEHnkhnWoS3NNeQEt_hEphNGCVbTFJFI9FekBHDHPEM66eMWQmfcfhvCfqu5V5GLG9dXaT2h_puZWolxSddyNp2UfujlV-pYvKaSf__MAXKqJRxk6HbWqEXgwIU37o8RMTySdB5pRVGJaqEqwbapUb6dQaUQtn_0dN9TJvWjBB6ZrnNjpyTs1OCeh48SKzGV-H_AnogThNDSG_TubpFjoQyqwj6zf6o4xAO4Ne1p7yNvDE_6q3gPrpE2/https%3A%2F%2Fgithub.com%2Fhwm2746%2Fvirion-spike-model
Codes in this link also include data generation for statistical analyses in Fig 6C,D, Supplemental Fig 14, and Supplementary Fig 15C,D.

-DOI: 10.5281/zenodo.8287823. https://secure-web.cisco.com/1frkoud-jn7Dx3E0TucoFgtaaxRXFPLQ1j9VMb7cXAsh0JAiGi_x8tegxeKbe4x93l7iAbCcVITLPZwLiI9ly_cPhpiA-EqOFHMuC3SvbkdcSFzZYYb82nxJEULxGC-QdZsfPmUn3OYUnPCd32yH03T3ILR7mwsKKREaVkrqzZVBeuvYJupZVycvFPGbCS75y55X8cNQkcE-q7FVpSJb2rOIm2vZUz4Nl7W7D4BdLA3PE445IuK5Ji07sSAjZicHupg810z90ZicF1H_F0wT6RQC8M3Y3Xk8B7YKWQeSaVuLtLsu7Z8tmvrLTTWZhh1PRSIBthtD4o76Lu6CGzYgq2g/https%3A%2F%2Fdoi.org%2F10.5281%2Fzenodo.8287823

-C++ source code and Phython programs to produce models in Fig. 6B and distributions in Fig. 6C,D and Supplementary Fig. 14 are available for download from https://github.com/hwm2746/virion-spike-model . Models and data for Supplemental Fig. 15B-D can be obtained by

modifying input parameters in this code, as explained in README.md file in the GitHub repository.

-Flow cytometry: cells were acquired and analyzed by using BD LSR Fortessa (BD Biosciences)
- Antibody binding kinetics were acquired by Biacore 3000 (Cytiva)

Data analysis

-HKL3000 program suite was used for data processing, including intensity integration, scaling and merging (Supplementary Table 1). Structures were determined by using molecular replacement method (Supplemntary Table 1). The final models were refined using the program Phenix.refine (Supplementary Table 1). Structural validation of each model was performed using the program MolProbity.

-Rendering of molecular structures and virion models in Fig 6A,B and Supplemental Fig 15A,B were done using VMD (https://secure-web.cisco.com/1PW8kIBWv-SuoBSmD4DFhLB539U28ehXnWK9YAE9bSNZ7937mp3KXq8SnVmp2yTCf-i3sGP15grVV3noDd7JgB0KZTIgtkdHWig1F1B8GDQOyHXsLXG6TNh9xzN30_FrkX5x_NW5UallnyLQQ7R__3389msS6Ln_oWccf1Koc7owHf7XNCMCeTK_HymrRwjHCmp4gidE-imwXYyYDyO7yotzI2L5CjbvchTA78kYVvFr7hgGvr-5MD9ARzZoQxShv_Td-eliqcFkp0k-iVWQVdGqVn3jErH8B0IDe5xiq21vzARc7gnaQ5qehDnYlcogo/https%3A%2F%2Fwww.ks.uiuc.edu%2FResearch%2Fvmd%2F). Plotting of distributions in Fig 6C,D, Supplemental Fig 14, and Supplemental Fig 15C,D was done using Python. The Python codes are also included in the GitHub link given in Data Collection.

-Flow cytometry data were analyzed using FlowJo software (BD Biosciences).
-Prism8 (Graphd) was used to calculate EC50/IC50 values on the ELISA and neutralization experiments.
-Biacore evaluation 3.1 software was used to determine KD values of antibodies in Biacore 3000 experiments.
-Image Lab software (Bio-Rad) was used to analyze SDS-PAGE gel.

For manuscripts utilizing custom algorithms or software that are central to the research but not yet described in published literature, software must be made available to editors and reviewers. We strongly encourage code deposition in a community repository (e.g. GitHub). See the Nature Portfolio guidelines for submitting code & software for further information.

Data

Policy information about availability of data

All manuscripts must include a data availability statement. This statement should provide the following information, where applicable:

- Accession codes, unique identifiers, or web links for publicly available datasets
- A description of any restrictions on data availability
- For clinical datasets or third party data, please ensure that the statement adheres to our policy

Atomic coordinates and structure factors for the reported crystal structures have been deposited in the Protein Data Bank under accession numbers: 8FWF, 8FYM, 8FXJ and 8FZ2, and all data are available in a publicly accessible repository

Research involving human participants, their data, or biological material

Policy information about studies with human participants or human data. See also policy information about sex, gender (identity/presentation), and sexual orientation and race, ethnicity and racism.

Reporting on sex and gender	N/A
Reporting on race, ethnicity, or other socially relevant groupings	N/A
Population characteristics	N/A
Recruitment	N/A
Ethics oversight	N/A

Note that full information on the approval of the study protocol must also be provided in the manuscript.

Field-specific reporting

Please select the one below that is the best fit for your research. If you are not sure, read the appropriate sections before making your selection.

☒ Life sciences ☐ Behavioural & social sciences ☐ Ecological, evolutionary & environmental sciences

For a reference copy of the document with all sections, see nature.com/documents/nr-reporting-summary-flat.pdf

Life sciences study design

All studies must disclose on these points even when the disclosure is negative.

Sample size	Sample size was generally chosen based on preliminary data indicating the variance within each group and the differences between groups.
Data exclusions	No data were excluded from the analyses.
Replication	All experiments were repeated as indicated in the manuscript, and where applicable we have now indicated the number of independent replications. Independent experiments yielded comparable results which were aggregated and subjected to statistical significance testing.
Randomization	No. As the goal of experiments was to directly compare gp160 binding by IgG1 vs IgG3 subtype of vaccine-elicited antibodies randomization was not relevant for in vitro experiment.
Blinding	No. it would be unfeasible to perform experiment/flow cytometry in a blinded manner.

Reporting for specific materials, systems and methods

We require information from authors about some types of materials, experimental systems and methods used in many studies. Here, indicate whether each material, system or method listed is relevant to your study. If you are not sure if a list item applies to your research, read the appropriate section before selecting a response.

Materials & experimental systems

n/a	Involved in the study	
☐	☒	Antibodies
☐	☒	Eukaryotic cell lines
☒	☐	Palaeontology and archaeology
☐	☒	Animals and other organisms
☒	☐	Clinical data
☒	☐	Dual use research of concern
☒	☐	Plants

Methods

n/a	Involved in the study	
☒	☐	ChIP-seq
☐	☒	Flow cytometry
☒	☐	MRI-based neuroimaging

Antibodies

Antibodies used

Monoclonal anti-HIV-1 Env 4E10
Monoclonal anti-HIV-1 Env 2F5
Monoclonal anti-HIV-1 Env 10E8
Monoclonal anti-HIV-1 Env Z13e1
Monoclonal anti-HIV-1 Env m66.6
Monoclonal anti-HIV-1 Env germline 4E10
Monoclonal anti-HIV-1 Env germline 2F5
Monoclonal anti-HIV-1 Env germline 10E8
Monoclonal anti-HIV-1 Env VRC01
Monoclonal anti-HIV-1 Env 460
Monoclonal anti-HIV-1 Env 235
Monoclonal anti-HIV-1 Env 275
Monoclonal anti-HIV-1 Env 274
Monoclonal anti-HIV-1 Env 484
Monoclonal anti-HIV-1 Env 203
Goat Anti-Human IgG-PE (Southern Biotech Cat # 2040-09, Lot # E0922-ZC42)
Goat Anti-Human IgG (H+L)-HRP Conjugate (Bio-Rad; Cat # 1721050)
Goat Anti-mouse IgG (H+L)-HRP Conjugate (Bio-RadCat #;1706516)

Validation

Flow cytometry study, ELISA and Biacore binding assays and neutralization assays performed in this study validated the function of all antibodies. The antibodies that are purchased from commercial sources (Southern Biotech and Bio-Rad) were validated by the manufacturers and documented in the manufacturer's website.

Eukaryotic cell lines

Policy information about cell lines and Sex and Gender in Research

Cell line source(s)	HEK293T (ATCC Cat# CRL-3216, RRID: CVCL_0063)

	HeLa-derived TZM-bl (NIH AIDS Reagent Program Cat# 8129-442, RRID: CVCL_B478)<br>Expi293F (Fisher Scientific, Cat# 13479756)
Authentication	HEK293T cells, TZM-bl cells and Expi293F cells were prepared sterilely in multiple aliquots from validated sources (NIH ARRRP, Thermo Fisher) and then stored under liquid nitrogen. Cell lines were passaged at the recommended frequency and seeding density in fresh media, and were routinely inspected under a microscope in the lab. Cells were discarded when the recommended passage number was reached.
Mycoplasma contamination	Cell lines were tested and found to be negative for mycoplasma contamination.
Commonly misidentified lines<br>(See ICLAC register)	No commonly misidentified cell line were used in this study

Animals and other research organisms

Policy information about studies involving animals; ARRIVE guidelines recommended for reporting animal research, and Sex and Gender in Research

Laboratory animals	BALB/c mice (Taconic) : 8-10 weeks old. MIce were fed a standard chow diet and housed in a temperature -controlled room under a 12-hour light-dark cycle.
Wild animals	No wild animals were used in the study
Reporting on sex	Female
Field-collected samples	No field collected samples were used in the study
Ethics oversight	All animal studies were approved by the Dana-Farber Cancer Institute and Harvard Medical School Animal Care and Use Committee Institutional Review Board.

Note that full information on the approval of the study protocol must also be provided in the manuscript.

Flow Cytometry

Plots

Confirm that:

- [x] The axis labels state the marker and fluorochrome used (e.g. CD4-FITC).
- [x] The axis scales are clearly visible. Include numbers along axes only for bottom left plot of group (a 'group' is an analysis of identical markers).
- [x] All plots are contour plots with outliers or pseudocolor plots.
- [x] A numerical value for number of cells or percentage (with statistics) is provided.

Methodology

Sample preparation	293T cells (100,000 cells) expressing HIV-1 gp145 or gp160 were washed in FACS buffer (PBS supplemented with 2% heat-inactivated FBS, 1 mM EDTA and 0.1% sodium azide) and stained with monoclonal antibody. After another wash, cells were stained with PE-conjugated goat anti-human IgG (Southern Biotech)mixed with the Zombie Aqua live/dead fixable dye (BioLegend) for the exclusion of dead cells. Soluble CD4 was incubated with cells for 1hr prior to staining cells with primary antibody.
Instrument	BD LSR Fortessa (BD Biosciences)
Software	FlowJo software 10.8.1 (BD Biosciences)
Cell population abundance	*Describe the abundance of the relevant cell populations within post-sort fractions, providing details on the purity of the samples and how it was determined.*
Gating strategy	PE+ and Zombie Aqua-(Provided in source Data)

- [x] Tick this box to confirm that a figure exemplifying the gating strategy is provided in the Supplementary Information.
